# First report of *Sarcoptes scabiei* parasitism (Sarcoptiformes: Sarcoptidae) in *Lycalopes sechurae* (Mammalia: Carnivora)

**DOI:** 10.1590/S1984-29612022036

**Published:** 2022-07-15

**Authors:** Ricardo Villalba-Briones, Eliana Belen Molineros, Juan Salvador Monrós

**Affiliations:** 1 Facultad Ciencias de la Vida, Escuela Superior Politécnica del Litoral – ESPOL, Polytechnic University, Campus Gustavo Galindo, Guayaquil, Ecuador; 2 Cavanilles Institute for Biodiversity and Evolutionary Biology, Universidad de Valencia, Paterna, Spain; 3 Fundación Proyecto Sacha, Guayaquil, Ecuador

**Keywords:** Sarcoptes scabiei, Lycalopex sechurae, mange, fox, wild trade, zoonosis, Sarcoptes scabiei, Lycalopex sechurae, sarna, raposa, comércio silvestre, zoonose

## Abstract

We present the first report of parasitism by *Sarcoptes scabiei* (Linnaeus, 1758), in a sechuran fox “*Lycalopex sechurae”*. *Sarcoptes scabiei* is a mite that produces sarcoptic mange, which can lead to the death of the animal host and can cause epidemic episodes in wildlife communities. The sechuran fox was collected by the environmental police from a citizen who reported the animal. It was sent to a veterinarian specializing in wildlife, “Clinica Mansion Mascota”, in Guayaquil, Ecuador. Immediate physical examination showed crusts on its skin, and samples of skin and blood were collected and analyzed. The skin samples were analyzed using a microscope at 40x magnification in the clinic. In addition, skin and blood samples were sent to a private laboratory for further analyses. Both analyses were positive for *S. scabiei* infection. It is the second report of *S. scabiei* in a new wildlife species in the Guayas province of Ecuador within less than a year. These events cause concern due the possibility of biological community transmission. Since domestic and feral animals are considered habitual spreaders of this disease, management through ethical procedures such as adoption, medical treatment and neutering campaigns, and awareness-raising projects with empathetic approach are recommended.

## Resumo

Apresentamos o primeiro relato de infecção por *Sarcoptes scabiei* (Linnaeus, 1758) em uma raposa-do-deserto-Peruana *Lycalopex sechurae*. *Sarcoptes scabiei* é um ácaro que produz sarna sarcóptica, que pode levar à morte do animal hospedeiro e causar episódios epidêmicos em comunidades de vida selvagem. A raposa-do-deserto-Peruana foi recolhida pela polícia ambiental de um cidadão que denunciou o animal. O animal foi enviado para a Clinica Mansion Mascota em Guayaquil, Equador, para ser examinado por um veterinário especializado em vida selvagem. O exame físico imediato mostrou crostas na pele, e amostras de pele e sangue foram coletadas e analisadas. As amostras de pele foram examinadas na clínica usando um microscópio com aumento de 40x. Além disso, amostras de pele e sangue foram enviadas para um laboratório privado para análises posteriores. Ambas as análises foram positivas para infecção por *S. scabiei*. Este é o segundo relato de *S. scabiei* em um novo hospedeiro selvagem na província de Guayas, no Equador, em menos de um ano. Isto indica que existe transmissão comunitária biológica. Uma vez que animais domésticos e feroses são considerados disseminadores habituais dessa doença, recomenda-se o manejo por meio de procedimentos éticos, como adoção, tratamento médico e campanhas de castração, e projetos de conscientização com abordagem empática.

## Introduction

Mange is a worldwide disease produced by various ectoparasitic mites (Rowe et al., 2019; Escobar et al., 2022). In the superfamily *Sarcoptoidea*, 12 families, more than 160 genera, and more than 1000 species affect various mammal and bird species (Bochkov, 2010). Among these mites, *Sarcoptes scabiei* (Linnaeus, 1758) generates the sarcoptic mange, an infectious disease which is considered an emerging global disease and a threat for biodiversity conservation (Escobar et al., 2022). Sarcoptic mange has a high prevalence in the tropics and large cumulative morbidity for human populations (Karimkhani et al., 2017). Coevolution has generated diverse specialization that drives mites to infect certain groups of species (Bochkov, 2010). *Sarcoptes scabiei* infects humans, domestic animals and wild species belonging to more than 16 families in ten of therian mammals, including primates, rodents, procyonids and canids, among others (Bochkov, 2010). Accompanying the success in the colonization of domestic animals, sarcoptic mange has become an essential threat for health of wildlife populations (Sepúlveda et al., 2014; Rowe et al., 2019). Transmission of sarcoptic mange can occur through direct contact or indirect contact. Direct contact takes place through allogrooming, while mating or fighting. Alternatively, indirect contact occurs when a healthy animal uses the space where an infected animal has made contact, for example, through infected dens, resting areas, or burrows (Escobar et al., 2022). Because of their abundance and feral habits, dogs, cats, and cattle had come into contact with wildlife, or had use the habitats of wild species (Sepúlveda et al., 2014; Zapata-Ríos & Branch, 2018). This, consequently, facilitates direct or indirect transmission of the disease (Sepúlveda et al., 2014; Rowe et al., 2019). The symptoms of sarcoptic mange include alopecia, hyperkeratosis and erythema, often accompanied by intense pruritus and loss of heat. Depending on multiple factors, these symptoms may be severe, and they produce weakening, loss of body mass and ultimately death (Süld et al., 2017; Villalba-Briones et al., 2022).

Recently, there have been reports of sarcoptic mange presence in wild and domestic animals in Guayaquil, Ecuador (Jordan et al., 2019; Villalba-Briones et al., 2022). In 2021 a white-nosed coati (*Nasua narica*) was captured in the Protected Forest of Cerro Blanco (Guayas, Ecuador) for treatment due to the high grade alopecia that showed to be a severe sarcoptic mange infection (Villalba-Briones et al., 2022). In this case *S. scabiei* had infested the 90% of its body surface and the individual perished 4 days after capture. Apart from this reports, few academical literature cover sarcoptic mange cases in animals that belong to the Ecuadorian wildlife, for example, the andean porcupine (*Coendou quichua*) in Colombia and the andean fox (*Lycalopex culpaeus*) in Chile (Gonzalez-Astudillo et al., 2018; Montecino-Latorre et al., 2020), but other countries, as Chile, Peru, Argentina, Bolivia and Brasil had addressed the problem of mange epidemics in several species (Montecino-Latorre et al., 2020). The majority of reports in South America belong to cases affecting domestic species such as rabbits (*Oryctolagus cuniculus*), dogs (*Canis familiaris*), cats (*Felis catus*), pigs (*Sus domesticus*), cattle (*Bos taurus*), llamas (*Lama glama*) and alpacas (*Vicugna pacos*) (Alcaino & Gorman, 1999; Bochkov, 2010; Gonzalez-Astudillo et al., 2018). It is also important to mention that from 1968 to 1973 a *S. scabiei* mange epidemic affected the human population in several localities from Ecuador (Carvajal et al., 1977; Karimkhani et al., 2017).

The sechuran fox, *Lycalopex sechurae* Thomas, 1900, is a relatively unknown solitary and nocturnal medium-sized canid (2.5-5.0 kg), distributed from northwestern Ecuador to central Peru (García-Olaechea & Hurtado, 2020). Its geographical distribution goes from the Sechura desert to the dry tropical forest of the Tumbesian region and the Marañon valley. In Ecuador, sparse data on this species presence in the coastal area are available from camera trap study reports (García-Olaechea & Hurtado, 2020). In Ecuador, sechuran foxes are not included in the Convention on International Trade in Endangered Species (CITES). Still, this species is considered to have a status of endangered in Ecuador (Tirira, 2021) and is protected through article 35 of the Ecuadorian Organic Environmental Code (Ecuadorian Environmental Organic Code, 2017).

Human behavior related to species overexploitation, habitat loss and degradation, exotic species introduction, global toxification, and climate change, are recognized factors that increase the likelihood of emerging of infectious diseases (EIDs) (Rush et al., 2021). Other factors influencing EIDS are high population densities, high agricultural land use, intensive livestock and poultry production, domestic animals, and, perhaps, greater biodiversity (especially of mammals) (Allen et al., 2017; Rush et al., 2021). Additionally, anthropogenic impacts, and, the presence of feral and domestic animals, are important sources of threat to the health of wild animal populations (Belsare & Gompper, 2015; Rush et al., 2021). Domestic animals pose a threat to wild animals because of their capacity to kill them, physically harm them, displace them from their natural habitats and transmit diseases (Rowe et al., 2019; Rush et al., 2021). In addition, capture, movement, and trade of wild species form a source of disease among wild and domestic species and can cause zoonotic episodes (Allen et al., 2017). Furthermore, several bacteriological, viral, endoparasitic, and ectoparasitic diseases have been linked to illegal trade of wildlife species and more abundantly from tropical areas (Rush et al., 2021). Captive birds, amphibians, reptiles, and mammals transmitted diseases to local wildlife, domestic animals, and human populations (Rush et al., 2021). Various anthropogenic impacts have been identified through studies in wildlife care centers in collaboration with environmental authorities (Verdugo et al., 2016). Such studies may lead to more efficient conservation actions.

This report presents the first case of sarcoptic mange in a sechuran fox and the second published case of mange affecting wildlife in the Guayas province and Ecuador (Villalba-Briones et al., 2022).

## Methodology

According to a report filed by the National Environmental Police Unit (UPMA: acronym in Spanish of Unidad Nacional de Policia Ambiente), a female juvenile sechuran fox was collected from a private housing development within the municipality of Duran, in Ecuador (2°13' 17.553” S, 79°42'6.086” W). This housing development is located apart from the urban area of Duran, at about 5 km to the east and near to the E-40 highway, surrounded by an area of disrupted tropical dry forest that has been heavily transformed by cattle farming and rice crops. This sechuran fox was found and collected by a citizen who stated that he kept it in his home for eight weeks. Thanks to collaboration from the environmental policy of the government of Ecuador, the sechuran fox was received in Mansion Mascota for diagnosis and implementation of treatment. Mansion Mascota is a private veterinary clinic that has a permit for conducting clinical treatment and rehabilitation on wild animals from the Environmental Ministry of Ecuador.

Upon arrival, the animal was sedated with intravenous ketamine (2 mg/kg) and propofol (2 mg/kg) for examination. Small crusty and alopecic areas on the ears and paws typical of mange infection were identified. Therefore, five skin samples were collected from edge of the lesion, from obviously pruritic areas through scraping, using a scalpel deep enough to cause capillary breakage (Kandi, 2017). Two of the samples were conserved in wax and added to the acarological collection of INABIO (acronym in spanish for National Biodiversity Institute of Ecuador) (access number INABIOEC-MECN-ACR-68). Further two samples were mounted and examined under a binocular microscope at 40x amplification (Kandi, 2017. Additionally, a set of samples (five blood samples, 1 ml, collected from the cephalic vein, and one skin sample) was sent to a private laboratory for hematological, pathological, cytological, bacterial, and viral analyses (REDLAV, Guayaquil, Ecuador).

Additionally, a private laboratory analyzed the condition of the skin samples and the level of mite infection through direct observation under a microscope. Pathogenical and bacteriological tests were also performed on skin samples. At the same time, because mange had previously been detected, accompanying other diseases in native fauna, PCR analysis was performed at a private clinic to test the samples for canine distemper virus (CDV) and an immunologic microELISA test for *Brucella canis* (REDLAV, Guayaquil, Ecuador). These analyses were negative. Hematological analysis showed a chronic inflammatory process and a stress leukogram (López-Villalba & Mesa-Sánchez, 2015). In addition, substantial bacteriological infections consisting of *Coccoides* sp. and *Bacillus* sp. were identified in skin samples. Hematological analyses showed absence of the hemoparasites *Anaplasma platys*, *Microfilaria* and *Babesia*. Although the laboratory did not divulge the procedures due to commercial secrecy concerns, these analyses helped to focus the veterinary treatment on mange infection.

The animal received a weekly dose of subcutaneous ivermectin at 0.4 mg/kg for three weeks. This medication was selected due to its effectiveness against mange infections in wild animals (Rowe et al., 2019).

## Results

**Figure 1:**
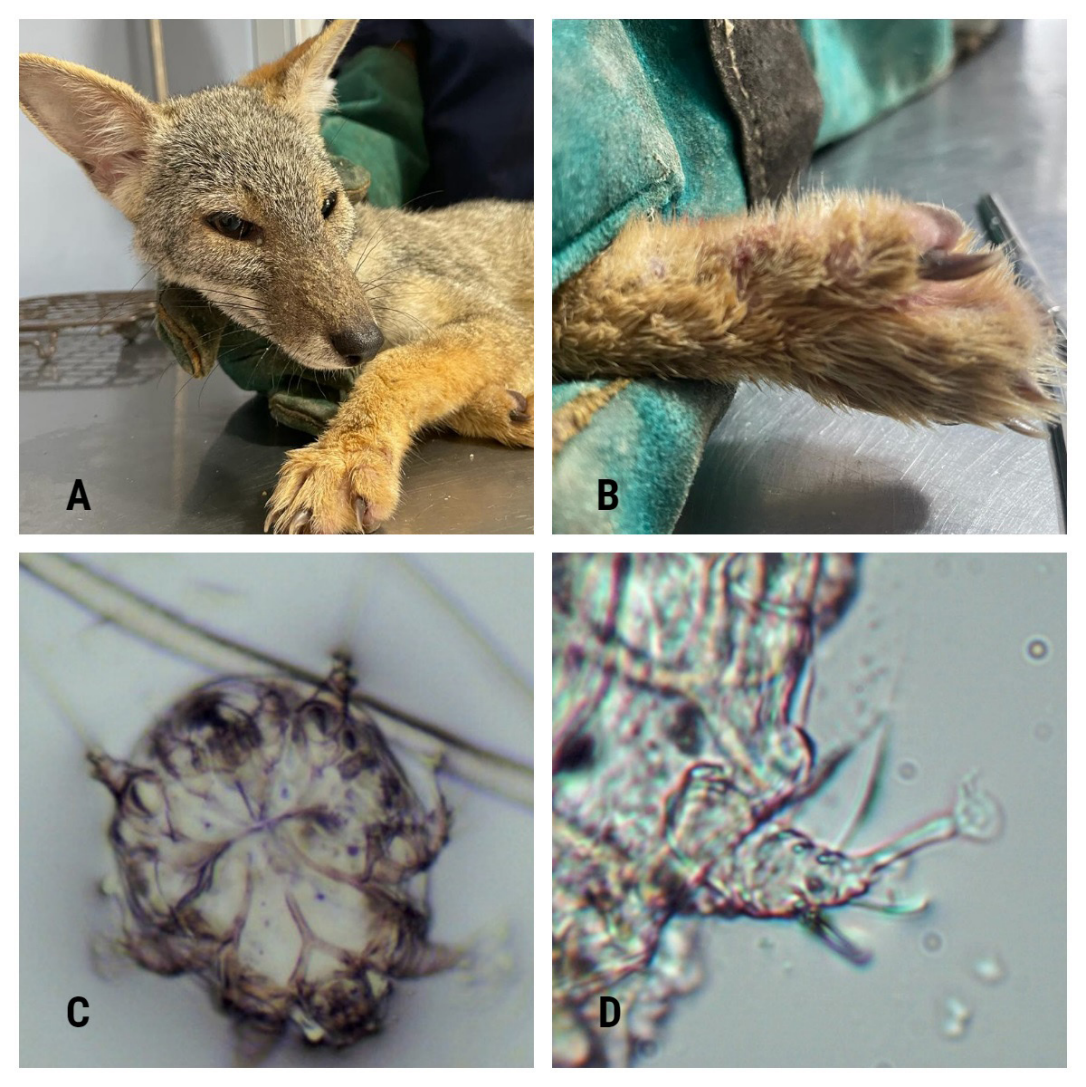
A. Sechuran fox at the stage of diagnosis. B. Sechuran fox paw image showing skin crusts. C. *Sarcoptes scabiei* image at 10x magnification, showing median apodeme fused. D. *Sarcoptes scabiei* leg IV showing fused tibia and tarsus at 40x magnification. Photo credit: Molineros E. B., Villalba-Briones R.

Upon admission, the diagnosis of the sechuran fox evidenced an scabies infection and a regular external clinical aspect regarding to the degree of muscle development, appearance of bone structure, hair and coat condition, presence of fatty deposits and face expression (Figure 1A) (Petersen et al., 2001). Less than 10% of the body surface of this sechuran fox (Figure 1A) was affected by clinical signs of scabies infection. Clinical signs observed included erythema and crusting on the epidermis (Figure 1B). After 3 weeks of treatment with ivermectin the body condition improved to good (Petersen et al., 2001). During the sixth week of treatment and care, the symptoms that had previously been diagnosed, i.e., erythema, alopecia, and scratching, disappeared.

*Sarcoptes scabiei* was observed in the epithelial tissue samples from the sechuran fox. Magnification of 40x facilitated observation of the characteristics of this mite species, as shown in the dichotomous key for the subfamilies and genera of Sarcoptidae (Klompen, 1992). Morphological examination of the mites (Figure 1C) showed typical characteristics of *S. scabiei*. Among other characteristics the idiosome was globose and elongated, the gnathostome was short and broad, and the legs were short and thick with fused tibia and tarsi IV (Figure 1D), and, the ventral median apodeme was fused with the genital sclerites (Figure 1C). Lastly, the anal shields were not fused to the posterior median shield (Figure 1C). Accordingly, the private laboratory indicated that *S. scabiei* was present in the skin samples. In addition, moderate levels of squamous cells, keratinocytes and cellular debris were identified.

## Discussion

The present report describes a second case of sarcoptic mange in wildlife in the same province of Ecuador at 11 months after the first case. Density of susceptible animals is related to the dispersion of this highly contagious disease. Environmental harsh conditions, climatic constraints, and food or water resources such as the periurban area where the here reported sechuran fox was collected, create common places for species use that increase the probability of exposure to sarcoptic mange (Sepúlveda et al., 2014; Escobar et al., 2022). Abandonment of dogs and a lack of fertility controls are common practices that lead to an abundancy of feral dogs populations that actively affect native carnivores in Ecuadorian Andes (Zapata-Ríos & Branch, 2018). Additionally, it is important to mention that feral dogs are a common problem in the Guayas province forested areas, dogs are more into contact with foxes than with other carnivores and their abundancy influence disease spillover events (Sepúlveda et al., 2014; Belsare & Gompper, 2015). Alternatively, mange is also present in housed dogs (Jordan et al., 2019) from which the sechuran fox could have been infected during captivity.

Campaigns to raise awareness of and promote animal welfare are strategies that have had the effect of instigating behavioral change in communities, towards wildlife conservation (Villalba-Briones et al., 2021). Inclusion of empathy-building strategies can lead to deeper understanding of the drama that these animals suffer and may produce altruistic motivation for protection of wild animals and their populations (Villalba-Briones et al., 2021). Furthermore, a higher social responsibility regarding to domestic and feral animals can be promoted, in order to minimize possible impacts towards wildlife (Sepúlveda et al., 2014; Zapata-Ríos & Branch, 2018).

## Conclusion

*Sarcoptes scabiei* poses a real threat to wildlife in the province of Guayas, Ecuador, and could impact the health of the populations of several species. Injected ivermectin was efficient in the treatment of this case of scabies infection in sechuran fox *L. sechurae*. This second case of sarcoptic mange within one year (Villalba-Briones et al., 2022) proves that mange is infecting various species. Sarcoptic mange can infect a wide array of species, and the continuous presence of dogs in natural habitats facilitates cross-species infection and spread. At the same time, illegally kept wild animals and their transportation, done by citizens, increases the rate of occurrence of infection among animals and the possibility of zoonotic episodes. Regarding to the possibility of mange outbreaks further research is needed to evaluate actual scabies prevalence in wildlife populations and establish whether this is an isolate case, or an individual victim of an outbreak. Knowledge of the diseases that occur in wildlife are important for management decision-making. Certainly, rehabilitation centers and protected areas collaboration could help monitoring zoonotic episodes. However, in addition to law enforcement, awareness-raising projects and diffusion of campaigns against wild trade and for stray dog population management are needed to decrease the numbers of detrimental activities towards wildlife. To reduce disease transmission, we recommend that an empathetic approach should be applied to this problem, based on promoting owner responsibility, neutering and medical treatment campaigns for feral and domestic animals.
